# Hindlimb venous distention evokes a pressor reflex in decerebrated rats

**DOI:** 10.14814/phy2.12036

**Published:** 2014-06-11

**Authors:** Katsuya Yamauchi, Audrey J. Stone, Marc P. Kaufman

**Affiliations:** 1 Penn State Heart & Vascular Institute, Pennsylvania State University College of Medicine, Hershey, Pennsylvania, USA

**Keywords:** Autonomic nervous system, dynamic exercise, peripheral artery disease, thin fiber muscle afferents

## Abstract

The distention of small vessels caused by an increase in blood flow to dynamically exercising muscles has been proposed as a stimulus that activates the thin fiber (groups III and IV) afferents evoking the exercise pressor reflex. This theory has been supported by evidence obtained from both humans and animals. In decerebrated unanesthetized rats with either freely perfused femoral arteries or arteries that were ligated 3 days before the experiment, we attempted to provide evidence in support of this theory by measuring arterial pressure, heart rate, and renal sympathetic nerve discharge while retrogradely injecting Ringer's solution in increasing volumes into the femoral vein just as it excited the triceps surae muscles. We found that the pressor response to injection was directly proportional to the volume injected. Retrograde injection of volumes up to and including 1 mL had no significant effect on either heart rate or renal sympathetic nerve activity. Cyclooxygenase blockade with indomethacin attenuated the reflex pressor response to retrograde injection in both groups of rats. In contrast, gadolinium, which blocks mechanogated channels, attenuated the reflex pressor response to retrograde injection in the “ligated rats,” but had no effect on the response in “freely perfused” rats. Our findings are consistent with the possibility that distension of small vessels within exercising skeletal muscle can serve as a stimulus to the thin fiber afferents evoking the exercise pressor reflex.

## Abstract (precis)

In decerebrated unanesthetized rats with either freely perfused femoral arteries or arteries that were ligated 3 days before the experiment, we attempted to provide evidence in support of this theory by measuring arterial pressure, heart rate, and renal sympathetic nerve discharge while retrogradely injecting Ringer's solution in increasing volumes into the femoral vein just as it excited the triceps surae muscles. Our findings are consistent with the possibility that distension of small vessels within exercising skeletal muscle can serve as a stimulus to the thin fiber afferents evoking the exercise pressor reflex.

## Introduction

Experiments in both animals and humans have provided strong evidence that at least part of the increases in arterial blood pressure and heart rate evoked by dynamic exercise arise from a reflex originating in contracting skeletal muscles (Friedman et al. 1993; Pickar et al. 1994; Adreani et al. 1997; Adreani and Kaufman 1998; Amann et al. 2010). The functional significance of this reflex, which has been named the exercise pressor reflex (Mitchell et al. 1983), lies in the fact that these cardiovascular effects have been shown to increase perfusion of exercising muscles (O'Leary et al. 1999; Amann et al. 2011). The sensory arm of the reflex evoking these increases is comprised of group III and group IV muscle afferents, of which the former were believed to be sensitive to mechanical stimuli and the latter sensitive to metabolic stimuli (McCloskey and Mitchell 1972; Kaufman et al. 1983). The exercise‐induced stimulus to group III mechanoreceptors has been frequently attributed to mechanical deformation of their receptive fields induced, in turn, by contractile elements in the muscle, whereas the exercise‐induced stimulus to group IV metaboreceptors has been frequently attributed to metabolic by‐products of contraction (Kaufman et al. 1983). This theory, which was based on electrophysiological findings, is consistent with anatomical evidence, which found that many of the endings of group III muscle afferents were located in connective tissue, whereas many of the endings of group IV muscle afferents were located in or near venules and small lymphatic vessels (Stacey 1969; von During and Andres 1990).

An alternative theory has surfaced suggesting that distention of small vessels within the exercising muscles caused by an increase in blood flow was the stimulus to activate the thin fiber afferents evoking the exercise pressor reflex (Huszczuk et al. 1993; Haouzi et al. 1995, 2004). This theory has been supported by evidence obtained from both humans and animals. For example, injection of vasoactive agents into the femoral artery of cats was found to stimulate group IV afferents innervating the resting triceps surae muscles (Haouzi et al. 1999). Likewise, retrograde injection of either saline or albumin into the brachial vein of an arm at rest was found to increase arterial pressure and muscle sympathetic nerve activity in humans (Cui et al. 2009, 2011).

In the experiments to be described we have examined the effect of retrograde injection of Ringer's solution into the femoral vein in evoking reflex pressor responses from skeletal muscles in decerebrated unanesthetized rats. Retrograde injection into the femoral vein distends venules and small lymphatics, raising the possibility that this stimulus can open mechanogated channels to stimulate group III and group IV afferents (Hayes et al. 2009). Likewise, retrograde injection can cause the release of ATP or cyclooxygenase metabolites of arachidonic acid, both of which can either stimulate or sensitize these thin fiber afferents (Rotto et al. 1990b; Hanna et al. 2002)**.** These findings prompted us to examine the effects of gadolinium, which blocks mechanogated channels, PPADS, which blocks Purinergic 2 receptors, and Indomethacin, which blocks cyclooxygenase, on the pressor reflex responses to retrograde venous injection.

We have also examined the effect of retrograde venous injection in both rats with freely perfused femoral arteries and in rats with femoral arteries that have been ligated for 3 days before the start of the experiment. We used the latter preparation for two reasons. First, ligation of the femoral artery for 3 days has been repeatedly shown to exaggerate the exercise pressor reflex (Tsuchimochi et al. 2010). Second, ligation of the femoral artery for this time period in rats has been shown to simulate the blood flow patterns to hindlimb muscle seen in peripheral artery disease. Specifically, blood flow to the muscles is normal at rest due to collateral circulation, but is greatly reduced and is inadequate to meet metabolic demand during treadmill exercise (Yang et al. 2000; Prior et al. 2004).

## Methods

All procedures were reviewed and approved by the Institutional Animal Care and Use Committee of the Pennsylvania State University, Hershey Medical Center. Adult male rats (Sprague‐Dawley, *n *=**90, weighing 438 ± 5 g) were used in this study. The rats were housed in a temperature‐controlled room (24 ± 1°C) with a 12:12‐h light–dark cycle. Rats were fed a standard diet and tap water ad libitum. Seventy‐two hours before an experiment, some of the rats underwent surgery to ligate the left femoral artery according to the procedure described previously (Tsuchimochi et al. 2010). Briefly, rats were placed into a sealed clear plastic box into which flowed a mixture of 4% isoflurane in 100% oxygen. After being removed from the box, the rats breathed the anesthetic gas mixture through a nose cone. The left femoral artery was isolated and then tightly ligated with 4–0 silk suture just distal to the inguinal ligament. In eight rats which served as sham controls for the ligation procedure, we exposed the femoral artery and passed the 4–0 silk suture under and through it. The rats were allowed to recover 72 h before the experiments were started. Femoral artery ligation has no effect on normal cage activity. Ligation has been shown to reduce blood flow reserve capacity to about 10–20% of normal but maintains sufficient blood flow to meet resting requirements (Yang et al. 2000; Prior et al. 2004). The rats which underwent the femoral arterial ligation procedure will be referred to as “ligated,” whereas those which did not undergo this ligation procedure will be referred to as “freely perfused.”

### Surgical preparation

On the day of the experiment, rats were anesthetized initially with a mixture of 4% isoflurane and 100% oxygen. The trachea was cannulated, and the lungs were ventilated mechanically (Harvard Apparatus) with 2% isoflurane in 100% oxygen. The right jugular vein and common carotid artery were cannulated (PE‐50) for the delivery of drugs and fluids and the measurement of arterial blood pressure, respectively. The carotid arterial catheter was connected to a pressure transducer (model P23 XL, Statham). Heart rate (HR) was calculated beat to beat from the arterial pressure pulse (Gould Biotach, Cleveland, OH). Arterial blood gases and pH were measured by an automated blood gas analyzer (model ABL‐700, Radiometer). Pco_2_ and arterial pH were maintained within 33–40 mmHg and 7.35 to 7.42, respectively, by either adjusting ventilation or by intravenous administration of sodium bicarbonate (8.5%). A rectal temperature probe was inserted and the core body temperature of the animal was maintained at 37–38°C by a water‐perfused heating pad and a lamp.

In each of the rats, we cannulated (PE‐10) the left femoral vein in a retrograde direction and advanced the tip of the cannula for 2–3 mm so that it was located just before the popliteal vein. These procedures allowed the retrograde injection of saline and other substances into the circulation of the left hindlimb. A snare was placed around the abdominal aorta and the inferior vena cava just above the aortic bifurcation. In freely perfused rats, a second snare was placed around the left femoral artery. When tightened, the snares helped to keep the injectate within the circulation of the left hindlimb. These procedures allowed the injection of substances directly into the circulation of the left hindlimb via the left femoral vein.

The rat was placed in a Kopf stereotaxic frame. Dexamethasone (0.2 mg) was injected intravenously just before the decerebration procedure to minimize brainstem edema. The left common carotid artery was tied off and a precollicular decerebration was performed. The plane of section was less than 1‐mm anterior to the superior colliculi. All neural tissue rostral to the section was removed, bleeding was controlled, and the cranial cavity was packed with cotton. Rats were decerebrated instead of anesthetized because the preponderance of the evidence indicates that anesthesia prevents the exercise pressor reflex in this species (Smith et al. 2001). Immediately after the decerebration, gas anesthesia was discontinued. The rats were allowed to stabilize for at least an hour. Before the start of the experiment the rats were paralyzed with pancuronium bromide (0.5 mg; IV) and their lungs were ventilated mechanically.

Using a retroperitoneal approach, we exposed bundles from the left renal sympathetic nerve. The bundles were then glued (Kwik‐Sil, World Precision Instruments, Sarasota, FL) onto a pair of thin stainless steel electrodes, which in turn were connected to a high impedance probe and then to an amplifier (model FT 10; Grass Instruments, Quincy, MA). Multiunit signals from the renal sympathetic nerve were filtered between 100 and 1 kHz and were displayed on a storage oscilloscope. At the end of each experiment, hexamethonium (20 mg/kg) was injected intravenously to abolish activity, thereby demonstrating that the activity was postganglionic in nature.

### Experimental protocols

#### Effect of venous distention

Volume trials were performed in 16 rats (eight ligated rats and eight sham rats). We examined the peak pressor and cardioaccelerator responses to retrograde injection of five volumes (i.e., 0.1 mL, 0.25 mL, 0.5 mL, 0.75 mL, and 1.0 mL) into the femoral vein. Injection volumes were made in ascending order. The highest volume of Ringer's injected, namely, 1 mL, was about 4% of the total volume of the rat hindlimb, which from measurements made in our laboratory was found to be about 20 mL. The percentage of total limb volume injected retrogradely into the femoral vein in the rats used in our experiments was almost identical to that used by Cui et al. (2011) in humans to evoke the venous distention reflex. Injection times varied between 3 and 18 seconds, with larger volumes taking longer. In each of the ligated and “sham” (i.e., freely perfused) rats, we tightened the snare placed around the abdominal aorta and inferior vena cava just before the femoral venous injection. In the “sham” rats, we also tightened the snare which was placed around the left femoral artery.

#### Effects of Indomethacin, Gadolinium, PPADS, and Lidocaine injection into the femoral vein

We examined the pressor and cardioaccelerator responses to retrograde injection of 1.0 mL saline into the left femoral vein before and 30 min after indomethacin (1 mg/kg), 60 min after gadolinium (10 mmol/L, 0.1 mL), 15 min after PPADS (10 mg/kg), and 10 min after lidocaine (2 mg, 0.1 mL), each of which was also injected retrogradely into the femoral vein. The different time intervals between the initial retrograde saline injections and the subsequent retrograde saline injections were the same as those shown in previous studies to achieve blockade (Rotto et al. 1990a; Hayes and Kaufman 2001; Stone et al. 2014). The snares were maintained for 10 min (Indomethacin), 10 min (Gadolinium and PPADS), and 5 min (Lidocaine), after which these were released. Indomethacin was dissolved in sodium carbonate (100 mmol/L) and diluted with saline so that dose of 1 mg/kg was administered (Rotto et al. 1990a). A 10 mmol/L solution of gadolinium trichloride (Aldrich) was dissolved in buffered HEPES (pH 7.3–7.45), after which a 0.1 mL was injected retrogradely into the left femoral vein (Hayes and Kaufman, 2001). PPADS was dissolved in saline and was injected retrogradely in a volume of 0.5 mL into the left femoral vein. In this and the following two protocols, none of the rats underwent the sham surgical procedure because this procedure could not explain the exaggerated pressor responses to retrograde injections of the two largest volumes in ligated rats (see Results). These rats were termed “freely perfused.”

#### Effect of Lidocaine injection into the jugular vein

We examined the pressor and cardioaccelerator responses to 1.0‐mL saline injection before and 10 min after Lidocaine (2 mg; 0.1 mL) was injected into the jugular vein. The snares were maintained for 5 min, after which these were released.

#### Effect of dorsal and ventral root transection

We performed a laminectomy to expose the lumbar and sacral portions of the spinal cord (L2–S3). In five rats, we measured the pressor and cardioaccelerator responses to retrograde femoral venous saline injection before and after transection from L2 to S3 left dorsal roots**.** In six others, we measured these responses to retrograde injection before and after transection of the L2 to S3 dorsal and ventral roots (Longhurst et al. 1980).

#### Measurement of venous pressure

In three freely perfused rats, we measured the pressure created in the femoral vein when we injected the five volumes of Ringer's solution. This was accomplished by inserting into the femoral vein two PE 10 catheters glued together side by side with Kwik‐Sil (WPI, Inc.). One catheter was used to inject Ringer's solution and the other was used to measure pressure.

#### Data analysis

Arterial blood pressure, heart rate (HR), and renal sympathetic nerve discharge were recorded with a Spike 2 data acquisition system (CED, Cambridge) and stored on a computer hard drive (Dell). Mean arterial pressure (MAP) is expressed in mmHg and HR in beats per minute (bpm). Baseline values for MAP and HR were determined immediately before contraction, which was not initiated until these values were stable. All values are expressed as means ± standard error of the mean (SEM). Statistical analyses of arterial pressure and heart rate for the “ligated” and “sham” rats were performed with a two‐way ANOVA with one repeated measure. When applicable, Holm–Sidak's multiple comparison post hoc tests were performed. Likewise, statistical analysis across the two groups was performed on the differences in mean arterial pressure and heart rate with a one‐way ANOVA. The criterion for statistical significance was set at *P *<**0.05.

## Results

**Figure 1.**
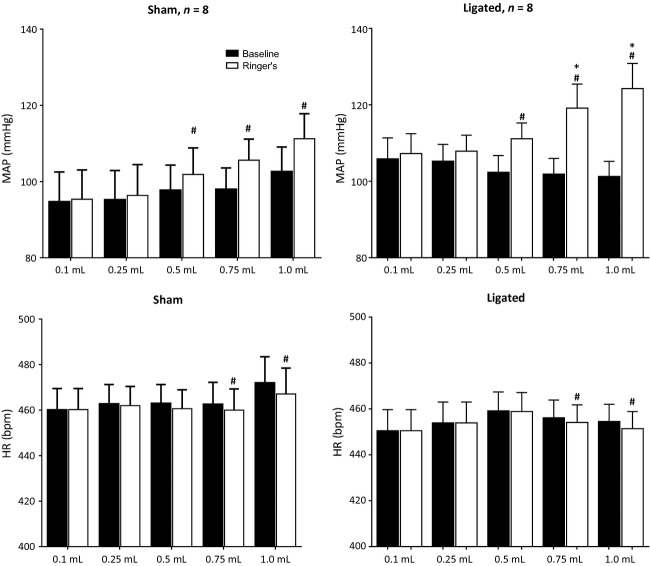
Effects of retrograde injection of five volumes of Ringer's solution (0.1 mL, 0.25 mL, 0.5 mL, 0.75 mL, and 1.0 mL) into the femoral vein on arterial blood pressure and heart rate in eight “sham” (i.e., freely perfused) rats and eight “ligated” rats. Filled bars represent baseline values and open bars represent peak values evoked by injection (mean ± standard errors). Abbreviations: MAP, mean arterial pressure. HR, heart rate. Number signs (#) depict significant difference (*P* < 0.05) between corresponding baseline and peak values before and after retrograde injection of Ringer's solution into the femoral vein. Asterisks (*) depict significant differences (*P* < 0.05) between the pressor responses to retrograde injection in “ligated” rats and the pressor responses to retrograde injection in “sham” rats for a given volume.

**Figure 2.**
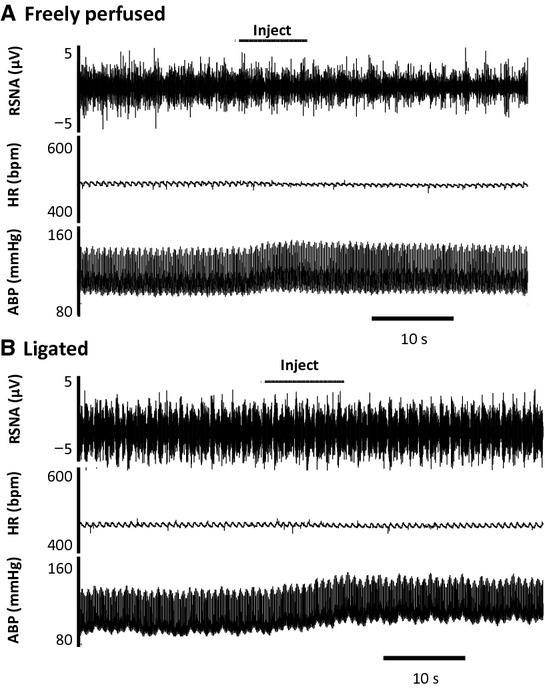
Individual records of the effect of retrograde injection of 1.0 mL of Ringer's solution into the femoral vein on renal sympathetic nerve activity (RSNA), heart rate (HR), and arterial blood pressure (ABP). Horizontal bar depicts the injection period. Note that the pressor response to injection is greater in the “ligated” rat (B) than it is in the “freely perfused” rat (A).

**Figure 3.**
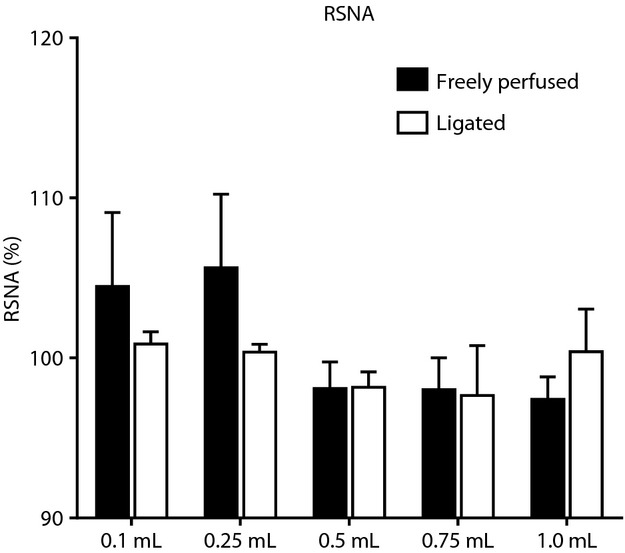
Effects of retrograde injection of five volumes of Ringer's solution (0.1 mL, 0.25 mL, 0.5 mL, 0.75 mL, and 1.0 mL) into the femoral vein on renal sympathetic nerve activity (RSNA) in eight “freely perfused” and “eight ligated” rats. Bars indicate changes from baseline values and represent means ± standard errors.

We found that retrograde injection of Ringer's solution into the femoral vein evoked a pressor response that was volume dependent in both the sham and the ligated rats (Fig. 1). The threshold volume needed to evoke a pressor response was 0.5 mL in both groups (Fig. 1). Moreover, the pressor response to retrograde injection of both 0.75 mL and 1.0 mL of Ringer's solution was greater in the ligated rats than it was in the sham rats (Figs. 1, 2). The injection volumes evoked only small decreases in heart rate in both groups. None of the injection volumes significantly changed renal sympathetic nerve activity in either the freely perfused or the ligated rats (Fig. 3). As a result, we stopped recording renal nerve discharge in the remainder of the experiments.

In three freely perfused rats, we measured the pressure developed in the femoral vein while we injected Ringer's solution. The pressure developed by the injection was high, but was uniform for the most part, across the five injection volumes (Table 1). The threshold volume needed to increase arterial pressure was 0.5 mL in both the sham and ligated rats.

**Table 1.** Peak femoral venous pressure evoked by injection of five volumes of Ringer's solution in three freely perfused rats. Note that the pressures were measured near the port of the cannula used to inject Ringer's solution

Volume (mL)	Venous pressure (mmHg)		Injection time (s)
	Baseline	Peak	
0.1	21.8 ± 4.4	103.9 ± 27.0	4.0 ± 0.4
0.25	19.3 ± 1.9	103.7 ± 24.3	6.6 ± 0.7
0.5	14.3 ± 2.3	99.3 ± 16.7	12.0 ± 1.8
0.75	16.7 ± 3.5	99.7 ± 17	12.7 ± 2.0
1.0	16.0 ± 1.5	106.7 ± 9.6	12.4 ± 2.4

**Figure 4.**
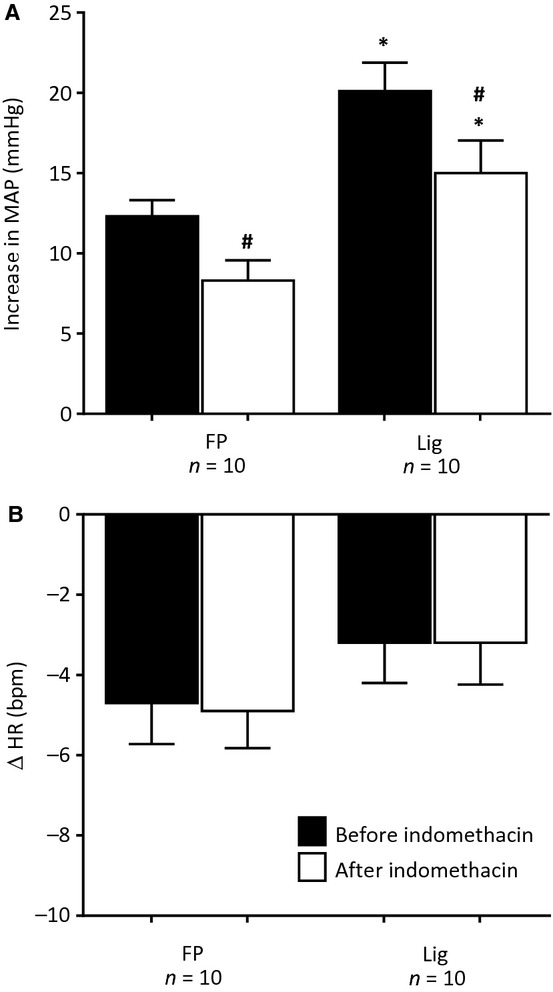
The pressor and heart rate responses to retrograde injection of Ringer's solution (1.0 mL) into the femoral vein before (filled bars) and after femoral vein injection of indomethacin (open bars) in 10 “freely perfused rats” (FP) and in 10 “ligated” (Lig) rats. Number sign (#) represents a significant difference (*P* < 0.05) between the pressor response before and after indomethacin. Asterisk (*) represents significant difference (*P* < 0.05) in the pressor response to injection between “freely perfused” and “ligated” rats.

We next examined the effects of indomethacin (1 mg/kg), retrogradely injected into the femoral vein, on the pressor response to retrograde femoral venous injection of Ringer's in 10 freely perfused rats and in 10 ligated rats. Indomethacin is a nonselective inhibitor of cyclooxygenase (COX) 1 and 2, the enzyme which converts arachidonic acid into prostaglandins and thromboxanes. In both groups of rats, indomethacin attenuated the pressor responses to injection of 1 mL of Ringer's (Fig. 4). In both groups of rats, retrograde venous injection of Ringer's had no significant effect on heart rate, a finding which was not altered by indomethacin.

**Figure 5.**
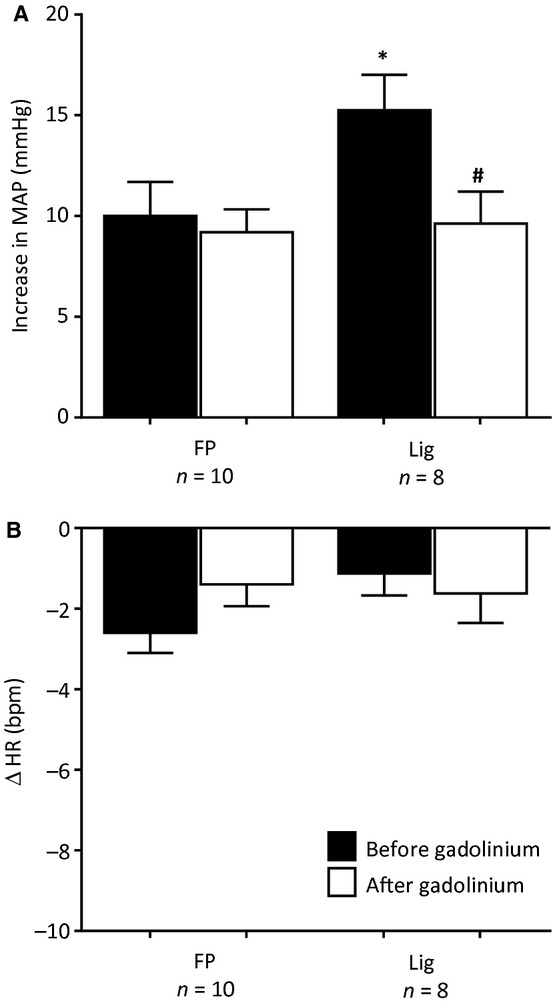
The pressor and heart rate responses to retrograde injection of Ringer's solution (1.0 mL) into the femoral vein before (filled bars) and after femoral vein injection of Gadolinium (open bars) in 10 “freely perfused” (FP) rats and in eight “ligated” (Lig) rats. Number sign (#) represents a significant difference (*P* < 0.05) between the pressor response before and after Gadolinium. Asterisk (*) represents significant difference (*P* < 0.05) in the pressor response to injection between “freely perfused” and “ligated” rats.

We examined the effects of gadolinium on the pressor responses to retrograde injection of 1 mL of Ringer's into the femoral vein in 10 freely perfused rats and in eight ligated rats. Gadolinium, which blocks mechanogated channels (Hayes and Kaufman, 2001), had no effect on the pressor response to saline injection in the freely perfused rats, but did attenuate this response in the ligated rats (Fig. 5). In both groups of rats, retrograde venous injection of Ringer's had no significant effect on heart rate, a finding which was not altered by gadolinium (Fig. 5).

The PPADS had no effect on the pressor response to retrograde venous injection of 1 mL of Ringer's solution in either the ligated or the freely perfused rats. Specifically, retrograde venous injection increased mean arterial pressure by 8 ± 1 mmHg both before and after PPADS in the freely perfused rats (*n* = 4; *P* = 0.32). Likewise, retrograde injection increased mean arterial pressure by 17 ± 3 mmHg before and after PPADS in the ligated rats (*n* = 4; *P* = 0.32).

**Figure 6.**
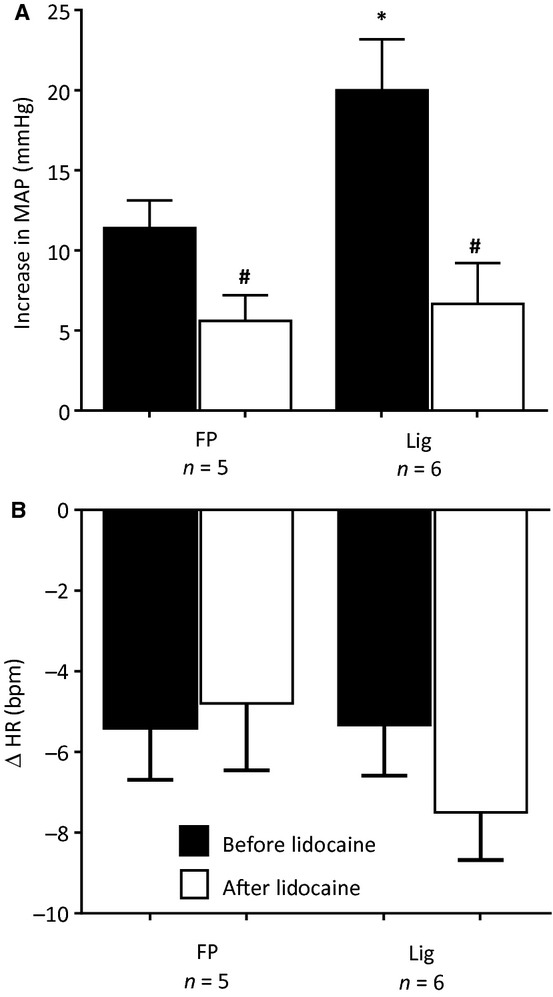
The pressor and heart rate responses to retrograde injection of Ringer's solution (1.0 mL) into the femoral vein before (filled bars) and after femoral vein injection of Lidocaine (open bars) in five “freely perfused” (FP) rats and in six “ligated” (Lig) rats. Number sign (#) represents a significant difference (*P* < 0.05) between the pressor response before and after Lidocaine. Asterisk (*) represents significant difference (*P* < 0.05) in the pressor response to injection between “freely perfused” and “ligated” rats.

In five freely perfused and in six ligated rats, we examined the effects of lidocaine (2 mg in 0.1 mL) on the pressor response to 1 mL of Ringer's solution, injected retrogradely into the femoral vein. In both groups of rats, the pressor responses to Ringer's solution were attenuated by retrograde injections of lidocaine into the femoral vein (Fig. 6). In both groups of rats, retrograde venous injection of Ringer's solution had no significant effect on heart rate, a finding which was not altered by lidocaine (Fig. 6).

**Figure 7.**
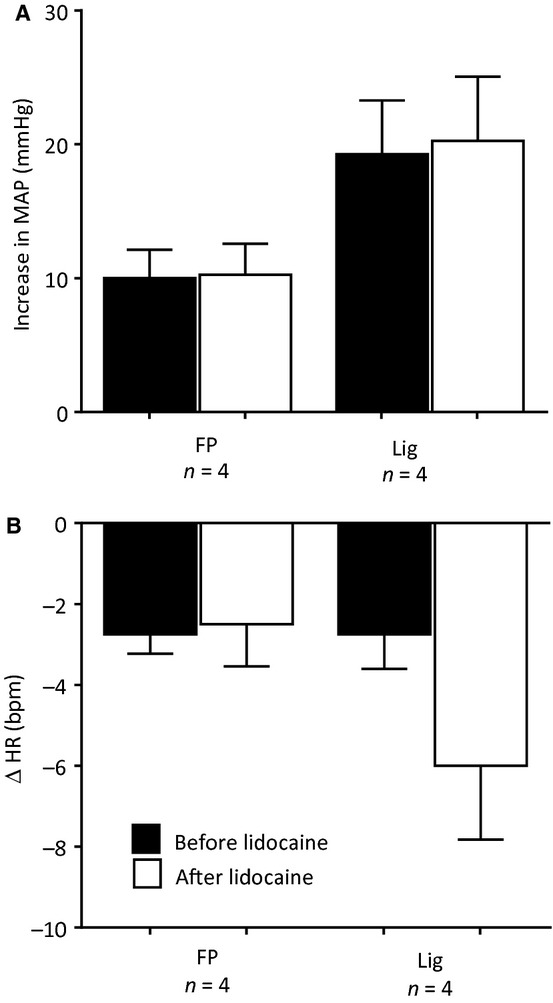
The pressor and heart rate responses to retrograde injection of Ringer's solution (1.0 mL) into the femoral vein before (filled bars) and after jugular vein injection of Lidocaine (open bars) in four “freely perfused” (FP) rats and in four “ligated” (Lig) rats. Note that lidocaine injected into the jugular vein had no effect on the pressor responses to retrograde injection of Ringer's solution in either the FP or the Lig rats.

To control for the possibility that lidocaine, injected retrogradely into the femoral vein, circulated to the spinal cord or brainstem, we injected this agent (2 mg in 0.1 mL) into the jugular vein, a maneuver which allowed it to circulate systemically. We found that in four freely perfused as well as in four ligated rats that the pressor responses to retrograde injection of Ringer's solution into the femoral vein were not attenuated by injections of lidocaine into the jugular vein (Fig. 7). In both groups of rats, retrograde venous injection of Ringer's solution had no significant effect on heart rate, a finding which was not altered by lidocaine (Fig. 7).

**Figure 8.**
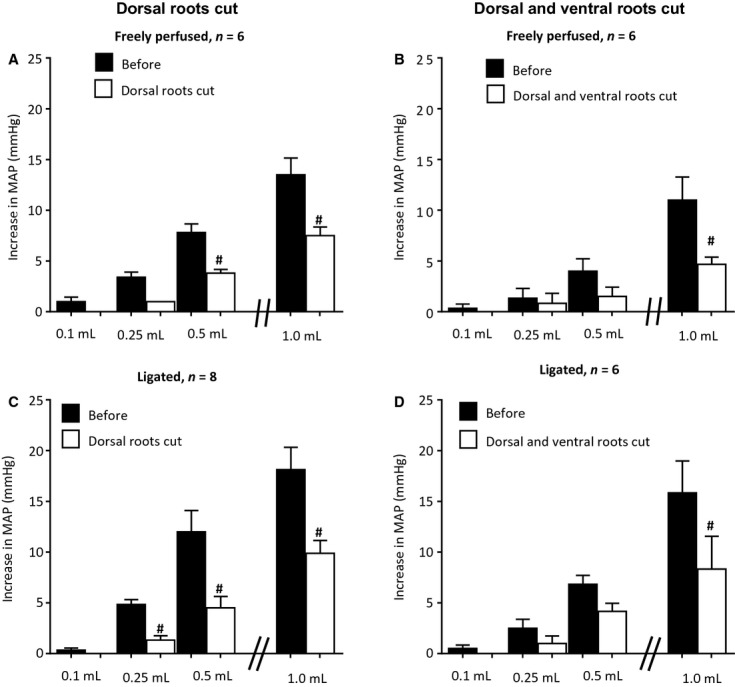
The effects of sectioning either the L2–S3 dorsal roots (A and B) or sectioning the L2–S3 dorsal and ventral roots (C and D) on the pressor responses to retrograde injection of four volumes of Ringer's solution into the femoral vein. Filled bars represent pressor responses before cutting and open bars represent pressor responses after cutting. A and C show data obtained from six “freely perfused” rats and B and D show data obtained from eight (B) and six (D) “ligated” rats. Note that rats having their dorsal and ventral roots sectioned were different than those having only their dorsal roots cut. Number signs (#) represent significant difference (*P* < 0.05) between the pressor response to retrograde injection before and after cutting the roots.

To confirm the reflex nature of the pressor responses to retrograde injection into the femoral vein, we measured the pressor responses to retrograde venous injection of several volumes of Ringer's before and after cutting the L2 to S3 left dorsal roots as well as before and after cutting the L2 to S3 left dorsal and ventral roots. Transection of the L2 to S3 dorsal roots in both freely perfused and ligated rats significantly attenuated the pressor responses to retrograde injection (Fig. 8). In addition, transection of the L2 to S3 dorsal and ventral roots in both groups did not appear to attenuate the pressor response to injection to any greater extent than did transection of only the dorsal roots (Fig. 8).

## Discussion

We found that in both freely perfused and ligated rats that retrograde injection of Ringer's solution into the femoral vein near its junction with the triceps surae muscles evoked a pressor response that in part was attributable to a reflex mechanism arising from distention of venules and possibly small lymphatics located within this muscle group. In contrast, retrograde injection of Ringer's solution had no effect on either heart rate or renal sympathetic nerve activity in either group of rats. A possible explanation for the lack of a cardioaccelerator response to retrograde injection was the high resting heart rate, which is often seen in our decerebrated preparation, and our use of pancuronium bromide, a vagolytic agent (Goodman et al. 2011). Likewise, the most likely explanation for the lack of renal nerve response to retrograde injection was that this particular sympathetic discharge is well established to be heavily under the control of the arterial baroreceptors**.**

In a few experiments, we measured pressure in the femoral vein draining the triceps surae muscles while we retrogradely injected Ringer’ solution into this vessel. We found that femoral venous pressure across all injection volumes was unphysiologically high but uniform. These findings led us to conclude that pressure alone was not sufficient to evoke a pressor reflex because the two smallest volumes, namely, 0.1 and 0.25 mL, did not evoke a reflex. Instead, we speculate that the simplest explanation for our findings is that the small intramuscular veins and lymphatics needed to reach a threshold volume in order to stimulate sufficiently the thin fiber afferents evoking a pressor reflex.

In our experiments, section of the dorsal roots from L2 through S5 or injection of lidocaine attenuated by at least half the pressor response to retrograde venous injection. Moreover, neither section of the dorsal roots alone nor section of both the dorsal and ventral roots abolished this pressor response. One explanation for the remaining pressor response after lumbar and sacral dorsal and ventral root section is that retrograde injection stimulated abdominal afferent endings innervated by axons traveling in the thoracic dorsal roots. Even though the femoral artery was occluded in our preparation, we cannot exclude the possibility that collateral vessels transmitted the injectate into the mesentery. This explanation may not be so farfetched. Recently, Garvin et al. (2014) reported that abdominal compression evoked pressor responses in humans which they speculated was reflex in origin. In addition, there is substantial evidence that stimulation of abdominal visceral mechanoreceptors innervating vessels evokes reflex pressor responses (Andrews et al. 1972; Nijima 1977). Moreover, afferent fibers innervating the small intestine have been shown to be stimulated by increases in mesenteric venous pressure (Andrews et al. 1972). A second and more likely explanation for the small pressor response to retrograde venous injection that remained after section of both the dorsal and ventral roots was that the large increase in pressure caused by the injection obstructed hindlimb blood flow.

Indomethacin, a cyclooxygenase antagonist which blocks the production of prostaglandins and thromboxanes, attenuated the reflex pressor responses to retrograde venous injection in both the freely perfused and ligated rats. Moreover, the magnitude of the attenuation appeared to be the same in both groups of rats. We think it reasonable to speculate that the distention caused by retrograde injection of Ringer's solution in our experiments activated phospholipase A2, which in turn, cleaved arachidonic acid from lipids in the membranes of cells comprising the intramuscular venules. Cyclooxygenase metabolites of arachidonic acid have been shown to either stimulate group III and group IV muscle afferents or to sensitize them to contraction. In addition, blockade of this enzyme has been shown to reduce both the exercise pressor reflex as well as the responses to contraction of group III and group IV afferents (Stebbins et al. 1988; Rotto et al. 1990a,b). Consequently, it would not be surprising if prostaglandin and thromboxane production induced by venous distention either stimulated these thin fiber afferents or sensitized them to this mechanical stimulus.

We found gadolinium injection restored the exaggerated pressor response to retrograde venous injection in ligated rats to the same level as that evoked by retrograde injection in freely perfused rats. Gadolinium, however, had no effect on the pressor response to retrograde venous injection in freely perfused rats. Gadolinium is believed to block mechanogated channels, and its injection into the arterial supply of skeletal muscle has been shown to significantly attenuate the exercise pressor reflex as well as the responses of group III mechanoreceptors to static and dynamic exercise (Hayes and Kaufman, 2001, Hayes et al. 2009). In addition, gadolinium has been shown to have no effect on the responses of group IV metaboreceptors to static or dynamic exercise (Hayes and Kaufman, 2001; Hayes et al. 2009). Likewise, it has been shown to have no effect on the responses of either group III or group IV muscle afferents to chemical stimulation (Hayes and Kaufman, 2001). Our findings with gadolinium suggest that the endings responsible for the exaggerated pressor response to retrograde venous injection in the ligated rats were mechanically sensitive, most of which are believed to be innervated by group III fibers. This suggestion may appear inconsistent with limited electron microscopic evidence that many of the endings of group III muscle afferents are located in or near connective tissue (von During and Andres 1990). Nevertheless, venules within skeletal muscle are known to have a sensory innervation (Stacey 1969), and it is conceivable that at least some of these are mechanically sensitive group III afferents.

PPADS, which blocks the purinergic 2X receptor, had no effect on the pressor response to retrograde venous injection in either group of rats tested in our experiments. The natural stimulus to P2X receptors is ATP, which is released by mechanical distortion, a stimulus which would be expected to occur in our experiments when we injected Ringer's solution. We note with interest that PPADS had minimal effect on the exercise pressor reflex in freely perfused rats, but attenuated the exaggerated reflex caused by femoral arterial ligation (Stone et al. 2014). Our findings with both PPADS and gadolinium in the present experiments raise the possibility that venous distension stimulates a different population of thin fiber muscle afferents than does contraction of muscles.

Substantial evidence exists showing that ligation of the femoral artery for 1–3 days before an experiment increases both the exercise pressor reflex as well as the proteins comprising ASIC3 and TRPV1 receptors in the soma of dorsal root ganglion cells innervating the hindlimb muscles (Xing et al. 2008; Liu et al. 2010). The role played by these receptors in causing the exaggerated pressor response to retrograde injection of Ringer's solution into the femoral vein is unclear and remains to be determined. We attached a low priority to investigating the role played by ASIC 3 and TRPV1 receptors in the present experiments using venous distention because they are thought to be involved mostly in signaling the spinal cord that blood/oxygen supply to exercising muscles is not adequate to meet demand. We did not contract muscles in the present experiments and therefore had no reason to suspect that receptors responding to metabolites would play a role in the reflex pressor responses evoked by retrograde venous injection of Ringer's. Our finding in ligated rats that gadolinium restored the pressor response to retrograde venous injection to the level found in freely perfused rats suggests that ligation of the femoral artery increases proteins forming mechanogated channels as well as proteins forming channels sensitive to metabolic by‐products of contraction. Unfortunately, the specific composition of gadolinium‐sensitive mechanogated channels is not known.

During dynamic exercise, intramuscular venules are distended and compressed by the rhythmic increase in blood flow to the working muscles, an effect which can be attributed to the muscle pump (Sheriff 2003). The rhythmic distension of small venules in exercising muscles might lead to increases in tension in the vessel walls, leading to a stimulation of thin fiber afferents. In our experiments, the purpose of injecting Ringer's solution retrogradely into the femoral vein was to simulate this distention to determine if it would evoke a pressor reflex response in decerebrated unanesthetized rats. We found that retrograde injection of Ringer's solution into the femoral vein evoked a pressor response, much of which was a reflex that can be attributed to mechanical stimulation of afferent endings in or near the venules in the triceps surae muscles. Cyclooxygenase metabolites of arachidonic acid were found to contribute to the stimulation of these distention‐sensitive thin fiber afferent endings located in or near intramuscular venules in both rats whose femoral arteries were freely perfused and in rats whose femoral arteries were ligated 3 days before the start of the experiment. However, mechanogated channels sensitive to blockade by gadolinium, which are found on group III afferents (Hayes and Kaufman, 2001, Hayes et al. 2009) did not appear to play a role in evoking the pressor response to venous distention in rats with freely perfused femoral arteries. This finding is consistent with a previous report showing that many more group IV afferents were stimulated by either venous distension or dilation (Haouzi et al. 1999).

In contrast, mechanogated channels may play a role in the exaggeration of the pressor response evoked by venous distention in rats whose femoral arteries were ligated 3 days before the experiment. We speculate that femoral artery ligation induced additional mechanogated channels on thin fiber muscle afferents innervating small vessels with the triceps surae muscles. Whether these new mechanogated channels are found on group III or group IV afferents remains to be determined. In any event, these newly formed mechanogated channels may play a role in causing some of the exaggerated exercise pressor reflex seen during treadmill walking in patients with peripheral artery disease (Baccelli et al. 1999).

## Acknowledgment

We thank Joyce Kim for her technical assistance.

## Conflict of Interest

No conflicts of interest are declared by the authors.

## References

[b1] Adreani C. M. Kaufman M. P. 1998 Effect of arterial occlusion on responses of group III and IV afferents to dynamic exercise. J. Appl. Physiol.; 84:1827-1833. 9609773 10.1152/jappl.1998.84.6.1827

[b2] Adreani C. M. Hill J. M. Kaufman M. P. 1997 Responses of group III and IV muscle afferents to dynamic exercise. J. Appl. Physiol.; 82:1811-1817. 9173945 10.1152/jappl.1997.82.6.1811

[b3] Amann M. Blain G. M. Proctor L. T. Sebranek J. J. Pegelow D. F. Dempsey J. A. 2010 Group III and IV muscle afferents contribute to ventilatory and cardiovascular response to rhythmic exercise in humans. J. Appl. Physiol.; 109:966-976. 20634355 10.1152/japplphysiol.00462.2010 PMC2963332

[b4] Amann M. Runnels S. Morgan D. E. Trinity J. D. Fjeldstad A. S. Wray D. W. 2011 On the contribution of group III and IV muscle afferents to the circulatory response to rhythmic exercise in humans. J. Physiol.; 589:3855-3866. 21646407 10.1113/jphysiol.2011.209353 PMC3171890

[b5] Andrews C. J. Andrews W. H. Orbach J. 1972 A sympathetic reflex elicited by distension of the mesenteric venous bed. J. Physiol.; 226:119-131. 4507800 10.1113/jphysiol.1972.sp009976 PMC1331156

[b6] Baccelli G. Reggiani P. Mattioli A. Corbellini E. Garducci S. Catalano M. 1999 The exercise pressor reflex and changes in radial arterial pressure and heart rate during walking in patients with arteriosclerosis obliterans. Angiology; 50:361-374. 10348424 10.1177/000331979905000502

[b7] Cui J. McQuillan P. Moradkhan R. Pagana C. Sinoway L. I. 2009 Sympathetic responses during saline infusion into the veins of an occluded limb. J. Physiol.; 587:3619-3628. 19470776 10.1113/jphysiol.2009.173237 PMC2742285

[b8] Cui J. Leuenberger U. A. Gao Z. Sinoway L. I. 2011 Sympathetic and cardiovascular responses to venous distension in an occluded limb. Am. J. Physiol. Regul. Integr. Comp. Physiol.; 301:R1831-R1837. 21940404 10.1152/ajpregu.00170.2011 PMC3233857

[b9] von During M. Andres K. H. 1990 35-41 *in*In: Zenker W. Neuhuber W. L. (eds.). Topography and ultrastructure of group III and IV nerve terminals of cat's gastrocnemius‐soleus muscle. The primary afferent neuron: A survey of recent morpho‐functional aspects New York Plenum

[b10] Friedman D. B. Brennum J. Sztuk F. Hansen O. B. Clifford P. S. Bach F. W. 1993 The effect of epidural anaesthesia with 1% lidocaine on the pressor response to dynamic exercise in man. J. Physiol.; 470:681-691. 8308749 10.1113/jphysiol.1993.sp019882 PMC1143941

[b11] Garvin N. M. Levine B. D. Raven P. B. Pawelczyk J. A. 2014 Pneumatic antishock garment inflation activates the human sympathetic nervous system by abdominal compression. Exp. Physiol.; 99:101-110. 24014806 10.1113/expphysiol.2013.072447

[b12] Goodman L. S. Brunton L. L. Chabner B. Knollmann B. C. 2011 Goodman & Gilman's pharmacological basis of therapeutics New York McGraw‐Hill

[b13] Hanna R. L. Hayes S. G. Kaufman M. P. 2002 alpha, beta‐Methylene ATP elicits a reflex pressor response arising from muscle in decerebrate cats. J. Appl. Physiol.; 93:834-841. 12183475 10.1152/japplphysiol.00237.2002

[b14] Haouzi P. Huszczuk A. Gille J. P. Chalon B. Marchal F. Crance J. P. 1995 Vascular distension in muscles contributes to respiratory control in sheep. Respir. Physiol.; 99:41-50. 7740211 10.1016/0034-5687(94)00083-c

[b15] Haouzi P. Hill J. M. Lewis B. K. Kaufman M. P. 1999 Responses of group III and IV muscle afferent fibers to distention of the peripheral vascular bed. J. Appl. Physiol.; 87:545-553. 10444611 10.1152/jappl.1999.87.2.545

[b16] Haouzi P. Chenuel B. Huszczuk A. 2004 Sensing vascular distension in skeletal muscle by slow conducting afferent fibers: neurophysiological basis and implication for respiratory control. J. Appl. Physiol.; 96:407-418. 14715672 10.1152/japplphysiol.00597.2003

[b17] Hayes S. G. Kaufman M. P. 2001 Gadolinium attenuates exercise pressor reflex in cats. Am. J. Physiol.; 280:H2153-H2161. 10.1152/ajpheart.2001.280.5.H2153 11299217

[b18] Hayes S. G. McCord J. L. Koba S. Kaufman M. P. 2009 Gadolinium inhibits group III but not group IV muscle afferent responses to dynamic exercise. J. Physiol.; 587:873-882. 19103679 10.1113/jphysiol.2008.164640 PMC2669976

[b19] Huszczuk A. Yeh E. Innes J. A. Solarte I. Wasserman K. Whipp B. J. 1993 Role of muscle perfusion and baroreception in the hyperpnea following muscle contraction in dog. Respir. Physiol.; 91:207-226. 8469845 10.1016/0034-5687(93)90100-o

[b20] Kaufman M. P. Longhurst J. C. Rybicki K. J. Wallach J. H. Mitchell J. H. 1983 Effects of static muscular contraction on impulse activity of groups III and IV afferents in cats. J. Appl. Physiol.; 55:105-112. 6309712 10.1152/jappl.1983.55.1.105

[b21] Liu J. Gao Z. Li J. 2010 Femoral artery occlusion increases expression of ASIC3 in dorsal root ganglion neurons. Am. J. Physiol. Heart Circ. Physiol.; 299:H1357-H1364. 20852050 10.1152/ajpheart.00612.2010 PMC2993190

[b22] Longhurst J. C. Mitchell J. H. Moore M. B. 1980 The spinal cord ventral root: an afferent pathway of the hind‐limb pressor reflex in cats. J. Physiol.; 301:467-476. 7411444 10.1113/jphysiol.1980.sp013218 PMC1279411

[b23] McCloskey D. I. Mitchell J. H. 1972 Reflex cardiovascular and respiratory responses originating in exercising muscle. J. Physiol.; 224:173-186. 5039977 10.1113/jphysiol.1972.sp009887 PMC1331532

[b24] Mitchell J. H. Kaufman M. P. Iwamoto G. A. 1983 The exercise pressor reflex: its cardiovascular effects, afferent mechanisms, and central pathways. Ann. Rev. Physiol.; 45:229-242. 6342515 10.1146/annurev.ph.45.030183.001305

[b25] Nijima A. 1977 Afferent discharges from venous pressoreceptors in liver. Am. J. Physiol.; 232:C76-C81. 189619 10.1152/ajpcell.1977.232.1.C76

[b26] O'Leary D. S. Augustyniak R. A. Ansorge E. J. Collins H. L. 1999 Muscle metaboreflex improves O_2_ delivery to ischemic active skeletal muscle. Am. J. Physiol.; 276:H1399-H1403. 10199868 10.1152/ajpheart.1999.276.4.H1399

[b27] Pickar J. G. Hill J. M. Kaufman M. P. 1994 Dynamic exercise stimulates group III muscle afferents. J. Neurophysiol.; 71:753-760. 8176437 10.1152/jn.1994.71.2.753

[b28] Prior B. M. Lloyd P. G. Ren J. Li H. Yang H. T. Laughlin M. H. 2004 Time course of changes in collateral blood flow and isolated vessel size and gene expression after femoral artery occlusion in rats. Am. J. Physiol. Heart Circ. Physiol.; 287:H2434-H2447. 15271665 10.1152/ajpheart.00398.2004

[b29] Rotto D. M. Hill J. M. Schultz H. D. Kaufman M. P. 1990a Cyclooxygenase blockade attenuates the responses of group IV muscle afferents to static contraction. Am. J. Physiol.; 259:H745-H750. 2118727 10.1152/ajpheart.1990.259.3.H745

[b30] Rotto D. M. Schultz H. D. Longhurst J. C. Kaufman M. P. 1990b Sensitization of group III muscle afferents to static contraction by products of arachidonic acid metabolism. J. Appl. Physiol.; 68:861-867. 2111312 10.1152/jappl.1990.68.3.861

[b31] Sheriff D. D. 2003 Muscle pump function during locomotion: mechanical coupling of stride frequency and muscle blood flow. Am. J. Physiol. Heart Circ. Physiol.; 284:H2185-H2191. 12742830 10.1152/ajpheart.01133.2002

[b32] Smith S. A. Mitchell J. H. Garry M. G. 2001 Electrically induced static exercise elicits a pressor response in the decerebrate rat. J. Physiol.; 537:961-970. 11744768 10.1111/j.1469-7793.2001.00961.x PMC2278979

[b33] Stacey M. J. 1969 Free nerve endings in skeletal muscle of the cat. J. Anat.; 105:231-254. 5802932 PMC1232131

[b34] Stebbins C. L. Maruoka Y. Longhurst J. C. 1988 Prostaglandins contribute to cardiovascular reflexes evoked by static muscular contraction. Circ. Res.; 59:645-654. 3545533 10.1161/01.res.59.6.645

[b35] Stone A. J. Yamauchi K. Kaufman M. P. 2014 Purinergic 2X receptors play a role in evoking the exercise pressor reflex in rats with peripheral artery insufficiency. Am. J. Physiol. Heart Circ. Physiol.; 306:H396-H404. 24285113 10.1152/ajpheart.00762.2013 PMC3920134

[b36] Tsuchimochi H. McCord J. L. Hayes S. G. Koba S. Kaufman M. P. 2010 Chronic femoral artery occlusion augments exercise pressor reflex in decerebrated rats. Am. J. Physiol. Heart Circ. Physiol.; 299:H106-H113. 20418475 10.1152/ajpheart.00141.2010 PMC2904125

[b37] Xing J. Gao Z. Lu J. Sinoway L. I. Li J. 2008 Femoral artery occlusion augments TRPV1‐mediated sympathetic responsiveness. Am. J. Physiol. Heart Circ. Physiol.; 295:H1262-H1269. 18660449 10.1152/ajpheart.00271.2008 PMC2544511

[b38] Yang H. T. Feng Y. Allen L. A. Protter A. Terjung R. L. 2000 Efficacy and specificity of bFGF increased collateral flow in experimental peripheral arterial insufficiency. Am. J. Physiol. Heart Circ. Physiol.; 278:H1966-H1973. 10843895 10.1152/ajpheart.2000.278.6.H1966

